# 

**Published:** 1854-01

**Authors:** 


### WESTERN JOURNAL

OF

### MEDICINE AND SURGERY.

NEW SERIES. JANUARY, 1854. VOL. I.—NO. 1.

EDITED BY

LUNSFORD P. YANDELL, M. D.

PROFESSOR OF PHYSIOLOGY AND PATHOLOGICAL ANATOMY, AND DEAN OF THE FACULTY OF THE UNIVERSITY OF LOUISVILLE.

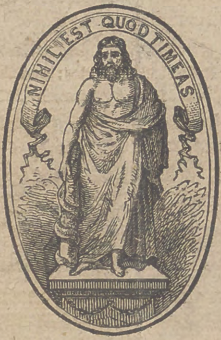

Surgeon Genl's Office
LIBRARY.
Washington, D.C.

LOUISVILLE, KY:

PRINTED AND PUBLISHED BY J. F. BRENNAN,

426 & 428 *Market Street.*

PRICE, $3 A YEAR—INVARIABLY IN ADVANCE.——POSTAGE, 2 CENTS A NUMBER.

